# Graduate and postgraduate educational challenges during the COVID-19 pandemic period: its impact and innovations—a scoping review

**DOI:** 10.1186/s13643-023-02359-2

**Published:** 2023-10-13

**Authors:** Muhammad Harris Shoaib, Muhammad Sikandar, Rabia Ismail Yousuf, Monica Parkash, Syed Jamil Hassan Kazmi, Farrukh Rafiq Ahmed, Kamran Ahmed, Muhammad Talha Saleem, Syeda Hina Zaidi

**Affiliations:** 1 Department of Pharmaceutics, Faculty of Pharmacy and Pharmaceutical Sciences, University of Karachi, Karachi, 75270 Pakistan; 2 Department of Geography, Faculty of Science, University of Karachi, Karachi, 75270 Pakistan

**Keywords:** COVID-19, Online education, PRISMA statement, Pandemic

## Abstract

**Background:**

The coronavirus disease 2019 (COVID-19) pandemic has transformed the global view of education, including graduate and postgraduate education making the development of an alternative approach in times of social isolation an academic imperative. The present review aims to investigate the challenges experienced among undergraduate and postgraduate education and the strategies adopted to address these challenges during the pandemic.

**Method:**

The preferred reporting items for the systematic review and meta-analyses extension for Scoping Reviews (PRISMA-ScR) were followed. The aim was to include journal articles published in the English language that discussed the influence of the pandemic on educational processes and applied innovative approaches as a solution to educational challenges. From January to August 2020, PubMed, EMBASE, and Google Scholar were searched for articles, yielding 10,019 articles. Two groups of authors examined the retrieved articles separately to avoid any risk of bias. The title and abstract of the articles were used for scrutiny, followed by full-text screening based on the established inclusion and exclusion criteria. The facts and findings of the studies were also discussed based on per capita income, literacy rate, and Internet accessibility.

**Results:**

Thirty of the obtained articles were included in the study. The selected articles were from North and South/Latin America, Asia & Pacific, South Africa, and Europe regions. Nineteen of the selected articles dealt with undergraduate education, ten with postgraduate, and one with both groups. The affordability of digital devices and the availability of Internet services were the major challenges for low- and middle-income economies. The ZOOM platform has been adopted by more than 90% of the education systems.

**Conclusion:**

Means of communication, including visual media, digitized content, and other web-based platforms, have been recognized as efficient learning and training tools, but have not been fully accessible for mass application and use due to the lack of availability of resources, their cost, and insufficient training among the users. In light of this review, it is suggested that harmonized and collaborative efforts should be made to develop cost-effective and user-friendly tools to overcome the current challenges and prevent future educational crises.

**Systemic review registration:**

The review was not registered.

**Supplementary Information:**

The online version contains supplementary material available at 10.1186/s13643-023-02359-2.

## Introduction

In late December 2019, a pneumonia-like disease caused by a novel coronavirus named severe acute respiratory syndrome coronavirus 2 (SARS-CoV-2) emerged in Wuhan, China. The disease was later named COVID-19. The disease spread worldwide and was declared a pandemic by the World Health Organization (WHO) in May 2020. Since no treatment options were available, strict preventive measures were recommended [1]. Visiting educational institutions and workplaces was banned, and both tourism and public gatherings were prohibited. In addition, citizens’ freedom of movement was restricted and controlled nationally and internationally to minimize and break the chain of infection [2, 3]. Normal life activities were disrupted and behavior and lifestyle changed enormously [4]. The education system has been one of the biggest casualties of the COVID-19 outbreak around the world, where the rate of spread was expected to be high due to the large and crowded gatherings and therefore closed as a priority [5]. Regular classroom activities were discontinued worldwide, and the continuity of education during the pandemic was one of the top concerns for each country [6, 7].

**Fig. 1:**
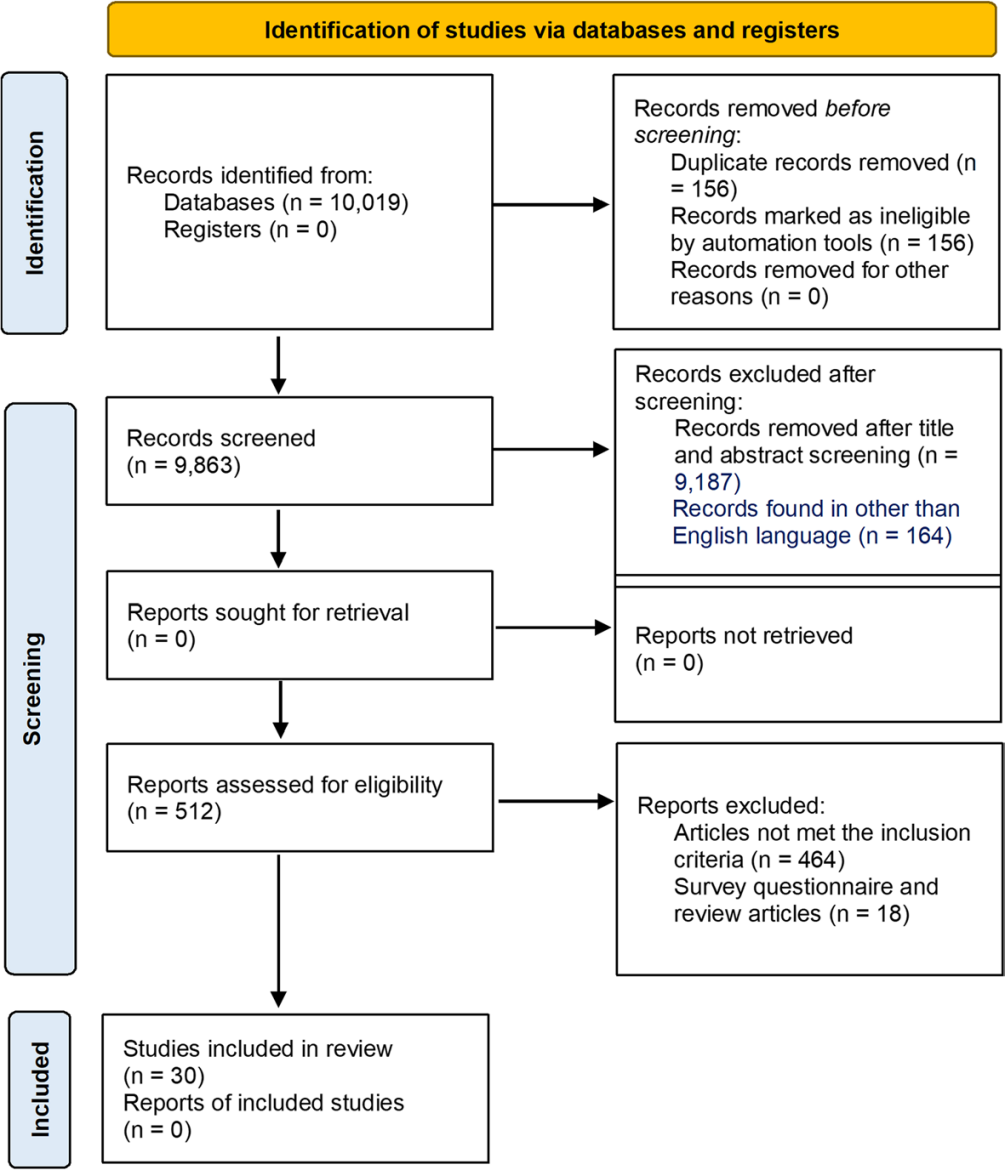
Flow diagram of the study selection [12]

**Fig. 2:**
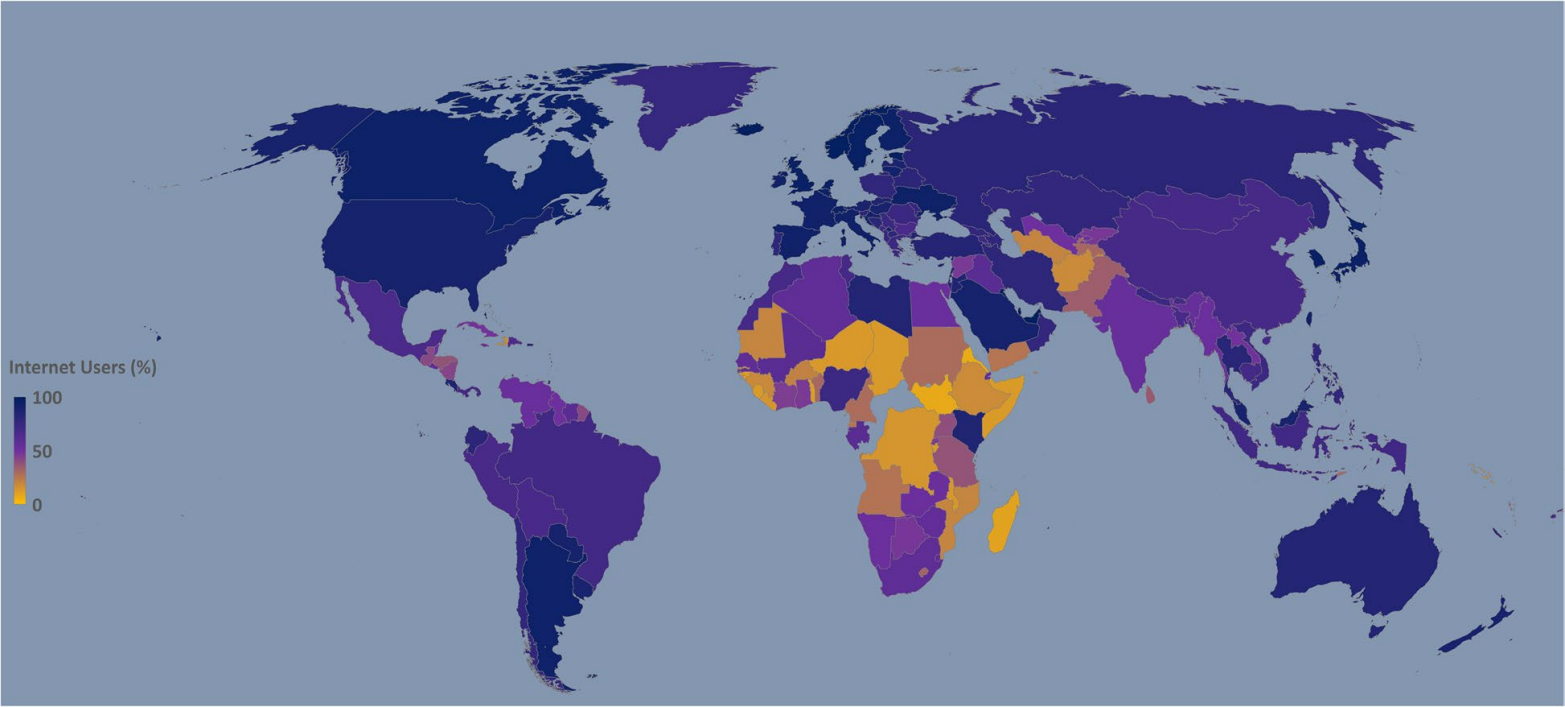
Representing the percentage of Internet users in different countries. Source: Internet World Stats Report (2021)

Distance learning or an online education system has been suggested by educators/government bodies as an effective alternative to face-to-face learning [6]. Various online teaching methods were adapted within a week or two, while some institutions opted for the hybrid educational model to complete the curriculum with the planned academic calendar. The exams were carried out via online platforms [8]. However, an uninterrupted Internet connection is the backbone for conducting online education. According to the United Nations International Children’s Emergency Fund (UNICEF) report published in 2017, more than half of the world’s population had no access to the Internet (Fig. 1) [9]. Considering the need, Internet accessibility has increased and Internet users are on the rise after the pandemic [10]. The latest information regarding Internet users country-wise is shown in Fig. 2 [11].

**Fig. 3:**
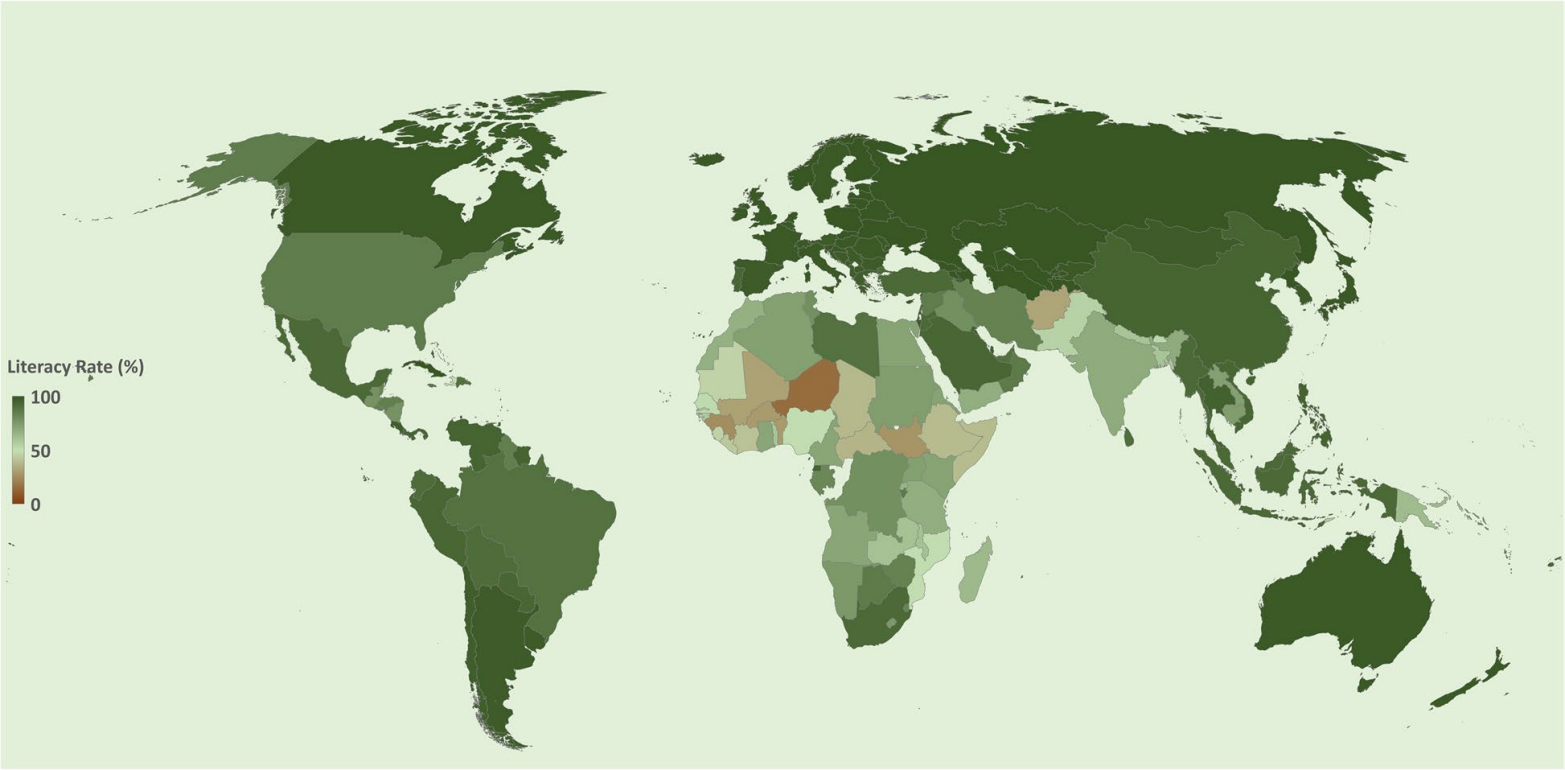
Representing the percentage of literacy in different countries. Source: WorldAtlas (2020)

Besides Internet accessibility, digital literacy is also an essential element required to embrace emerging technologies and communicate in today’s world [13]. Digital literacy is the ability to create, evaluate, communicate, integrate, understand, manage, and access information appropriately and safely through digital devices. These include media literacy, information literacy, information and communication technology literacy, and computer literacy [14]. Conversely, according to a 2016 report by the United Nations Educational, Scientific and Cultural Organization (UNESCO) Statistics Institute, nearly 750 million adults worldwide lack basic literacy skills. This is another major challenge for educating the masses who are overwhelmingly in the 15–24 age group. Some regions have the lowest reported literacy rate with 50% of the world’s illiterate population living in South Asia, followed by sub-Saharan Africa (~27%). East and Southeast Asia (10%), North Africa, West Asia (9%), and Latin America (4%) [15]. Shockingly, the report also pointed out that global literacy rates increased by just 4% between 2000 and 2015. The 2020 global literacy rate data provided on the World Atlas website is shown in Fig. 3 [16].

**Fig. 4:**
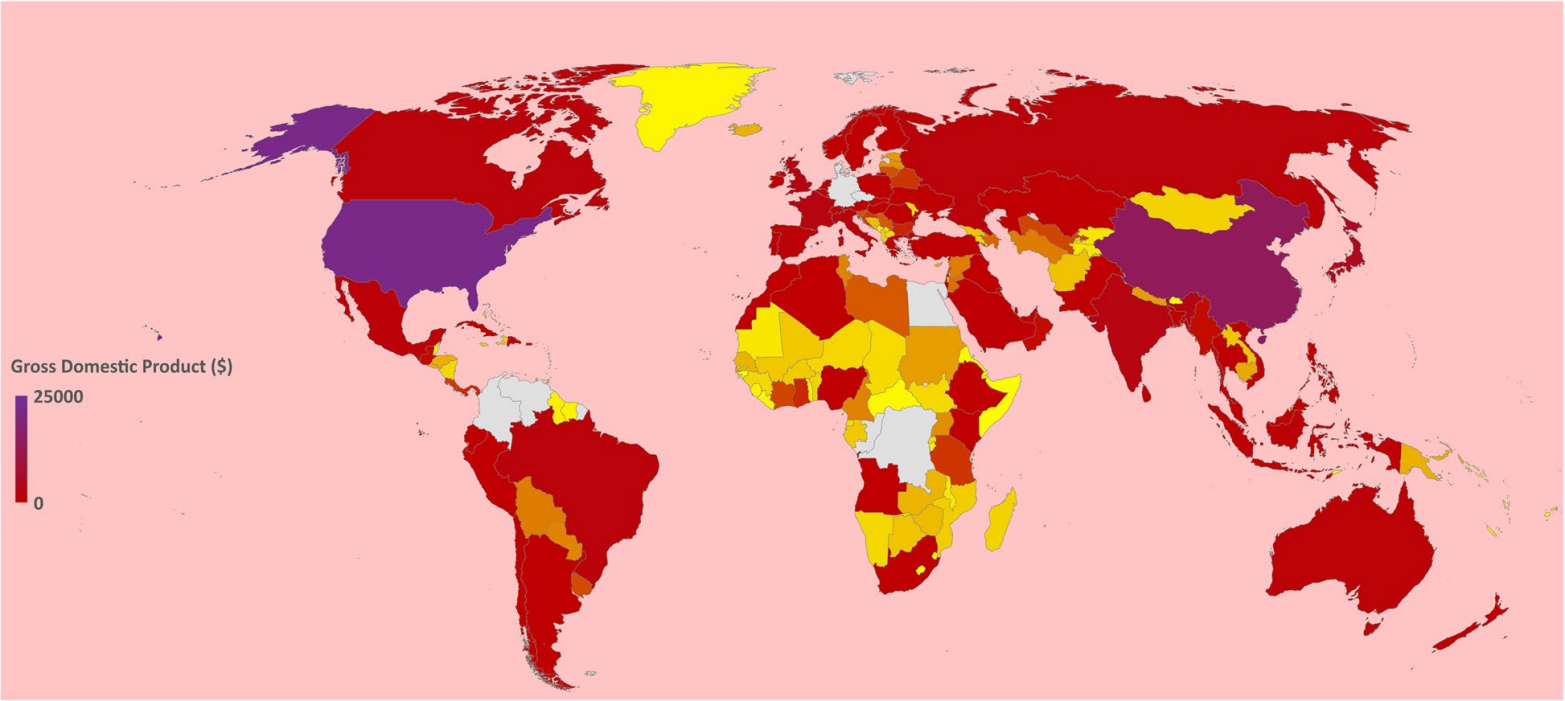
Representing the GDPs of different countries. Source: World Bank Report (2019)

**Fig. 5:**
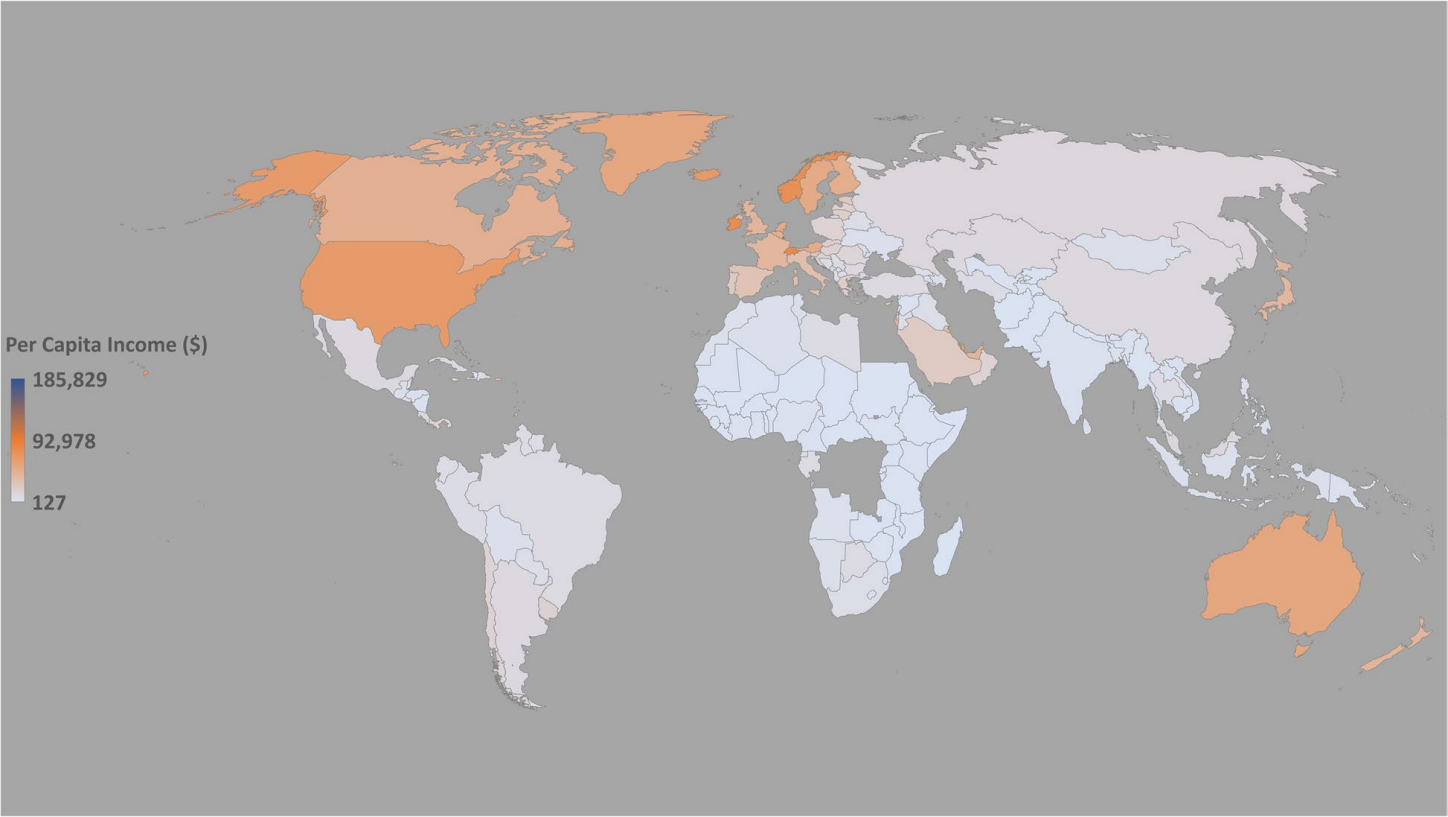
Representing the GDPs per capita of different countries. World Bank Report (2019)

In an old but very descriptive and conclusive study, Robert J. Barro demonstrated that there is a linear relationship between a country’s gross domestic product (GDP) and literacy [17]. The GDPs of different countries according to the 2019 World Bank report are shown in Fig. 4, showing the inequality between different continental regions [18]. In addition, the quality of the education provided is considered an essential element for national development and is therefore linked to GDP per capita [19]. The per capita income of different countries determines the technological purchasing power of the population to cope with online teaching methods (see Fig. 5 of the World Bank 2019 report) [20].

This urgent need and lack of resources for the adoption of distance learning amid the pandemic have severely impacted the entire education system around the world, particularly in developing countries where face-to-face (physical) interaction in the classroom is the traditional way of teaching [21]. When delivering content, meeting curriculum requirements is more difficult when there is a significant need for hands-on demonstration or laboratory work. In addition, it was observed that the theoretical aspects of the courses were being taught relatively at ease and with fewer difficulties as compared to the practical work [22]. According to a UNESCO report, this scenario has affected, in one way or another, almost a billion learners worldwide, particularly in the professional and higher education sectors [23].

In summary, the development of online learning, during the COVID-19 outbreak, presented a new challenge to the already ailing education system. The objective of the present review is to identify the major issues that have emerged in undergraduate and postgraduate education during COVID-19 and to identify the strategies that universities of different countries are adopting to address these challenges. In addition, the challenges and the importance of the implemented strategies are examined in the context of per capita income, literacy rate, and Internet availability in different parts of the world. This work offers possible alternative learning and teaching approaches to adopt during times of social distancing and school closures. The work also presents some suggestions to prevent such academic provocations in the future.

## Methodology

A systematic review of the literature was performed based on the Preferred Reporting Items for Systematic Reviews and Meta-Analyses extension for Scoping Reviews (PRISMA-ScR) reporting guidelines [24]. The PRISMA-ScR checklist can be found in supplementary Table S1. Because the study aimed to include articles related to the major education crisis during the COVID-19 pandemic, the time period was limited to January–August 2020. The PubMed, EMBASE, and Google Scholar (advanced search) databases were searched using the keywords “(‘coronavirus’ OR ‘SARS-CoV-2’ OR ‘COVID-19’) AND (‘graduate*’ OR ‘education*’ OR ‘teach*’ OR ‘train*’ OR ‘student*’ OR ‘challenge*’ OR ‘solution*’ OR ‘innovation*’)”. The search keywords were set to identify and recognize all the studies published on the challenges facing undergraduate and postgraduate education during the COVID-19 conditions and to cover the new methodologies being used as solutions to these challenges.

### Eligibility criteria

PCC (Population, Concept, and Context) was applied for the inclusion of sources in the scoping review [25]. For the current study, undergraduate or postgraduate students were selected as the test population. The concept of this review was to examine and identify the major educational issues that have arisen during COVID-19, along with possible alternative and widely accepted strategies adopted by various regional institutions to address the challenges. The outcomes were defined as responses to solutions or innovations used to address remote learning during the pandemic. In this context, the literature included research letters or articles, letters to the editors, and short communication in English, discussing the impact of the COVID-19 pandemic on undergraduate and postgraduate education and implementing innovative approaches as a solution to the challenges. Any literature deemed relevant according to the eligibility criteria was shortlisted for full-text screening based on the title and abstract. The review excluded the studies that did not discuss the challenges directly linked to the COVID-19 pandemic and did not apply any strategic solutions to address the education crisis at the undergraduate or postgraduate level. In addition, review articles, surveys or questionnaires, prospective pilot studies, books, book chapters, reports, papers based on primary and secondary education, and those published in a language other than English were also not included.

### Data collection and quality assessment of the studies

The selected studies were screened and evaluated for their eligibility using the predefined inclusion and exclusion criteria. The redundancy in the two databases was first identified using the EndNote application version X7.8. The first author provided the idea and fully directed the study. Articles retrieved were filtered into pairs by the second, fourth, eighth, and ninth authors independently on the basis of title and abstract. The articles whose eligibility was unclear were selected for a full-text review. These articles underwent further evaluation in a group of two independently by the second, third, sixth, and seventh authors, to ensure that the data from selected studies address challenges and the implementation of solutions or innovations in undergraduate or postgraduate education in the situations of the COVID-19 pandemic and prevent any risk of bias. Finally, those that met the predefined selection criteria were selected for data extraction.

The research articles selected for data extraction were reviewed and their key findings and facts were collected and sorted into a separate file. After completing the scrutiny of all articles, the findings were accumulated in two categories: [1] “challenges” and [2] “solutions or innovations.” The “solutions or innovations” have also been categorized into various secondary themes, namely teleconferences, webinars, virtual classroom and video conferences, new application or software design, social media platforms, simulation, and virtual reality. The strength of the adopted strategies (solutions or innovations) of the different online platforms used by different institutions was evaluated and the inference was made.

## Results

### Search outcomes and analysis

Out of 10,019 articles, the search items returned 8474 articles from PubMed, 1172 from EMBASE, and 373 from Google Scholar. After removing the duplicates, 9863 articles were obtained. A total of 512 articles were selected for a full-text review based on their title and abstract, and upon full-text screening, a total of 30 articles were selected and subjected to analysis for final data extraction. The articles were based on qualitative studies generally from the North American region followed by Asia and the Pacific, Europe, and South/Latin America. Figure 1 outlines a detailed description of the present research.

The two categories of the results, i.e., challenges and solutions/innovations with the secondary themes are described as follows.

### Challenges

Nineteen of the thirty articles addressed the challenges faced by undergraduate students, ten of the articles addressed postgraduate students, and one addressed both groups.

The cessation of all academic activities, reduced in-person educational opportunities, poor Internet connections, lack of technical understanding, limited resources, difficulties in assessment, pandemic-related stress, or anxiety were the most commonly reported educational challenges during the COVID-19 pandemic [26–32]. The reduction of hands-on learning and the cost burden of expensive online educational platforms available were the main challenges in undergraduate and postgraduate education [33–37].

### Solutions or innovations

#### Teleconferences, webinars, virtual classrooms, and video conferences

**Fig. 6:**
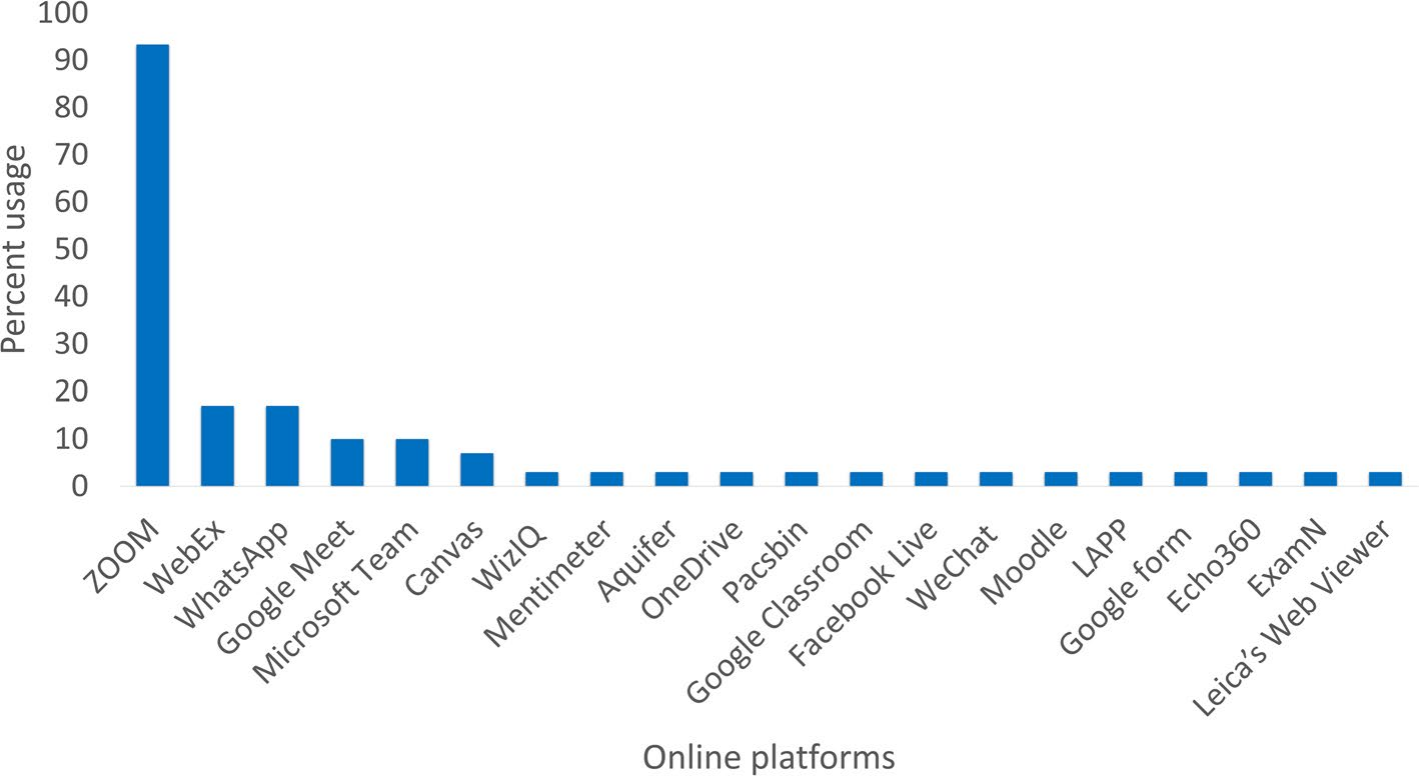
Percent use of online platforms during the COVID-19 period

The majority of the articles selected, around 23, are related to teleconferences, webinars, virtual classrooms, and video conferences (see Table 1). Most of these articles were from North America, generally the USA. ZOOM, WebEx, Microsoft Team, Mentimeter, Redmond, Wash, Pascsbin, Google Classroom, Echo360, GoToMeeting, Google Meet, and Adobe Connect received positive feedback as online learning tools in these regions [27, 28, 36–46]. Some of the articles were from Asia and Pacific, including countries such as India, Singapore, Japan, Korea, Pakistan, and China. ZOOM, Google Meet, Microsoft Team, and WebEx were reported as platforms in these regions [30, 31, 34, 47]. The UK and Ireland successfully adopted ZOOM, GoToMeeting, and EMIS Health [48, 49]. Likewise, ZOOM, Moodle, and WhatsApp were adopted in South Africa [50]. In South America, on the other hand, ZOOM, Google Classroom, Microsoft Team, and Athena Hub were used for online education [51]. In addition to the above platforms, social media platforms like Facebook Live, YouTube, WhatsApp, Snapchat, Telegram, Instagram, WeChat, and Twitter were leveraged to eliminate technology-based challenges. The hybrid education system (mixture of online and physical instruction), interactive online learning through face-to-face virtual meetings, and non-interactive online learning through the provision of recorded videos and supplemental reading materials were seen as the widely preferred approaches in the Asia Pacific region, where limited technology resources and insufficient knowledge to operate the advanced technologies were the main problems. ZOOM turned out to be the most used and widely preferred means of communication (Table 2). In the current review, more than 90% of the studies, indicated that ZOOM is an online platform preferred by both developed and underdeveloped countries (Fig. 6). Microsoft Teams, a video conferencing application similar to ZOOM, is also widely used. Meanwhile, telemedicine, in which patients are consulted online using telephone or video conferencing tools, has emerged as a widely accepted strategy for delivering medical education and remote consultation [41].

**Table 1:** Description of the articles categorized into teleconferences, webinars, virtual classrooms, and video conferences

**S. No.**	**Article type**	**Country**	**Region**	**Number of participants**	**Type of participants**	**Online platforms**	**Description of the adopted strategy**	**Outcomes**	**Strength of evidence**
**1**	Research letter (Singh & Arya, 2020) [31]	India	Asia & Pacific	200	Undergraduate students	ZOOM, Google Meet, Cisco WebEx, Microsoft Team, and WizIQ	Lessons were delivered through pre-recorded video and on live platforms using PowerPoint slides with audio, audio-visual with animated graphics, and audio-visual discussion.	Pre-recorded video lectures received good feedback.	Provide the least disturbance and flexibility of learning especially in regions where Internet connectivity is an issue.
**2**	Research letter (Pollom et al., 2020) [38]	USA	North America	12	Undergraduate students	ZOOM and WebEx	Virtual clinical clerkships in oncology were offered. Sessions on oncology were delivered through e-learning tools in week one to provide students a basic knowledge of the subject followed by a virtual demonstration of clinic patients and virtual student talks on published papers on the subject in week 2. Canvas web application was used to access ZOOM lectures and assignments. Meetings were also recorded and shared for review later by the students and ZOOM chat was used to engage students throughout the session. A post-clerkship assessment form was completed.	67 % of the participants found the program interested.	Provide a chance of clinical clerkship to medical students when all on-site clerkship programs are suspended. Moreover, increase student exposure to the oncology field and develop their interest in radiation oncology as a career.
**3**	Research article (Patterson, Ritwik, Kerins, & Adewumi, 2021) [39]	USA	North America	50	Postgraduate students	ZOOM and Mentimeter	Microsoft PowerPoint presentations were delivered virtually on the ZOOM platform to dentistry residents from three states. A real-time multiple choice question-based assessment was conducted on the cloud-based package Mentimeter Feedback was recorded anonymously and discussed in chat during the presentation.	A positive experience of learning was reported by the participants. Similarly, in an average of more than 80% of assessments, results were observed.	Participants from multiple states can be involved in the training session with a feature of real-time assessment and anonymous feedback. Moreover, involve of multiple institutions reduced the workload on individual university during COVID-19.
**4**	Research article (Durfee et al., 2020) [40]	USA	North America	111	Undergraduate students	ZOOM and Aquifer Modules	A 4-week interactive virtual radiology clerkship was designed which included an online flipped classroom model, large group lectures, and small group activities followed by online assessment through traditionally used multiple choice-based exams. Pre-reading materials were provided by Aquifer (Aquifer Modules). Large group didactic lectures and small group homeroom activities were conducted on the ZOOM platform. Student assessment via a standardized closed-book exam was performed, and feedback from these students was collected using online surveys. Moreover, a comparison of students’ performance was done to in-person radiology clerkship.	Scores of final exams were identical to in-person clerkships. An 85% mean exam result was observed, almost similar to the last 5-year traditional exam result which was in the average of 78%. The adopted strategy survey indicated that 98% of the participants found the structure of the course was excellent. The highest overall satisfaction for virtual homeroom activities was observed.	During this time of pandemic, a complete virtual radiology core clerkship can be a successful educational experience for medical students. The adopted small group learning environment was observed most successful method for engaging students and developing their interest in learning.
**5**	Report (Chowdhury et al., 2020) [36]	USA	North America	More than 3000	Postgraduate students	Teleconferencing and Videoconferencing town halls and the university’s official website (named “Care and Share”), OneDrive, Microsoft Team, Redmond and Wash	Teleconferencing applications were used to engage the students. Videoconferencing town halls for question answers and to receive feedback on current response measures were arranged. The “Care and Share” website was created that allowed the University of Washington Medicine community to connect with one another. Biweekly virtual happy hours were instituted that helped to maintain the sense of community. Furthermore, online tools such as Wash, Redmond, Microsoft Corp., and OneDrive were used to increase the productivity of the research.	The strategies adopted have been proven beneficial. Teleconferencing, videoconferencing, and other adopted programs were found helpful to continue residency and increased student’s engagement even after COVID-19 crisis.	These innovations can become an addition to the “new normal” routine of residency programs for the enhancement of learning and critical care.
**6**	Research Article (Abraham et al., 2020) [41]	USA	North America	20	Undergraduate Students	ZOOM	Online orientation was conducted on the ZOOM platform and students were trained to complete the American College of Physicians (ACP) module on telehealth. A survey based on the assessment of student’s attitudes, skills, and knowledge to telehealth at the end of the program was conducted.	Telemedicine was supposed to be an appreciated add-up in the medical field by 90% of the students. Overall satisfaction with the current online program was observed.	The program provides an alternative platform for medical students to experience clinical learning opportunities in the time of pandemic when in-person patient care is believed unsafe.
**7**	Research Article (McRoy et al., 2020) [37]	USA	North America	18	Postgraduate students	Pacsbin, ZOOM, and Google Classroom.	The radiology workstation was simulated by the implementation of a novel cloud-based distance learning solution. This suggested education model utilizes three tools i.e., Pacsbin, ZOOM, and Google Classroom. Pacsbin was used for case collection, case exchange, and case presentation. Students can easily access the cases uploaded to Pacsbin over the Internet and can be reviewed by the application’s Digital Imaging and Communications in Medicine (DICOM) viewer. A centralized classroom for assignments, reports, and discussion was created by using Google Classroom. ZOOM video conference readout was employed for the review of daily case collections in small groups.	Survey results from residents showed that following this project 78% of residents felt more prepared for the call, 78% were interested in the continuation of the project even after the pandemic, 78% were interested in extending this educational model in other specialties of radiology, and 78% found the caseload to be appropriate. Survey results from senior resident teachers revealed that the percentage of residents extremely interested in pursuing academic radiology increased from 43 to 57%.	Provided the ease of implementation of the described technological tools, this model has the tendency to be an excellent fit for any institution nationwide.
**8**	Research Letter (Maeda et al., 2020) [34]	Japan	Asia & Pacific	35	Postgraduate students	ZOOM	Endoscopic sinus surgery was instructed using PowerPoint lectures and videos on surgery on the Zoom application. Comments were acknowledged by chat or voice through the hand-raising method during and after the lecture.	The participants reported the adopted learning method was interactive or very interactive.	The use of online conferencing is beneficial not only for special circumstances but also in case of distance surgical education as it saves travel time and serves as an inexpensive mode of education.
**9**	Research Article (Parker, Chang, & Koch, 2020) [49]	UK	Europe	70	Undergraduate student	ZOOM	A comprehensive 2-week remote-learning course containing lectures, virtual slides, discussion groups, and unique case-based activities was developed. ZOOM meetings and chats were used to present lectures. ZOOM along with PowerPoint slides and PathPresenter with virtual slides was employed for morning didactics. Multiple strategies and technologies including screen annotation, “flipped classroom” slide presentations, and repetition of common themes were employed to increase engagement while distance learning	A nearly 10-fold increase in average pathology rotators and as well as positive attitudes toward pathology were observed.	The adopted method can be used to deliver lectures using an online platform.
**10**	Research article (Lee, Kim, Park, & Henning, 2021) [30]	Korea	Asia & Pacific	86	Undergraduate students	ZOOM platform	Assessment of the behavior of students for cheating fairness was performed on three technologies a tablet PC with a face-tracking option (face-tracking technology), ZOOM videoconferencing with side view of students face and posture, and random sequencing of questions test on a computer.	ZOOM platforms were found more effective technique to prevent cheating during the test and control cheating by 95% followed by random sequence questioning by 67%, and face tracking technology by 32%, respectively.	Provide a simple technology to control cheating and ensure fairness in the examinations.
**11**	Letter to the editor (Khan, 2021) [47]	Pakistan	Asia & Pacific	N/A	Undergraduate and postgraduate students	WhatsApp, ZOOM, Google Meet, and Facebook Live	Private groups were created for students from the same semester on Facebook, ZOOM, and Google Meet, and live sessions were delivered. Furthermore, the live sessions were also recorded and shared on the WhatsApp group of the same class to support revision.	The live sessions on Facebook were found more welcoming and suitable learning techniques with minimum resources while the added recorded lectures appeared beneficial to students with poor Internet connection issues.	Social networking and social media are found effective learning tools for developing countries.
**12**	Report (North, Vitto, Hickam, & Santen, 2020) [42]	USA	North America	N/A	Undergraduate students	Clinical reasoning and differential diagnosis sheet	Students were remotely linked with patients on cell phones, tablets, or other devices under the supervision of a resident. Students practiced history taking, differential diagnosis, clinical reasoning, and patient management skills. In the last, feedback from the students was taken.	The learning strategy has been found beneficial by the students which helped them to increase their clinical knowledge during the pandemic.	This design provided students an opportunity to practice real-world case studies from a remote setting in the present situation where practice in a direct clinical environment is not allowed.
**13**	Research Article (Chen, Kaczmarek, & Ohyama, 2020) [27]	USA	North America	39	Undergraduate students	ZOOM	Recorded video lectures were provided to the students. Live ZOOM polling was used to assess the perceptions and preferences of students related to recorded lectures and other course formats in post-course feedback sessions.	Students reported an overall worsening situation in learning after shifting toward e-learning. 44% of students responded the situation to be “somewhat worsened” and 26% responded as “significantly worsened.” Polling results showed an increased level of exhaustion, the same perceptions of attendance, decreased engagement and retention, a combination of pre-recorded lectures, recorded live lectures, and synchronous follow-up sessions were preferred and students were found to be similarly comfortable for both formats. Generally, students felt that more interactive virtual classes, like question-and-answer sessions and case-based small group discussions, would improve engagement.	A combination of synchronous and asynchronous modes of distance education for online future courses will improve student learning.
**14**	Research Article (Damien, Chappell, & van der Hoeven, 2021) [28]	USA	North America	N/A	Undergraduate students	WebEx and Canvas	Students were divided into small groups. Live sessions were delivered on WebEx and supplementary video lectures were provided on Canvas. At the end of the session, students were asked to submit a report comprised of three important lessons they learned. Furthermore, a quiz was also conducted on Canvas at the end of each session.	A positive feedback was received from the students. The use of multiple cameras, management of time, and order of lecture content were observed as important features of the successful delivery of online sessions.	The strategy could be the best alternative to simulation and hands-on learning during the period of social distancing.
**15**	Research Article (Guerandel, McCarthy, McCarthy, & Mulligan, 2021) [48]	Ireland	Europe	N/A	Undergraduate Students	N/A	A module based on live teaching, self-learning, tutoring, and peer support was presented. Workshops, small group tutorials, and Q & A sessions were conducted. Supplementary reading materials were provided along with tutor and peer review support through email and virtual meet up. In addition, the output of the students was also evaluated.	The learning approach was welcomed by students and trainers. It was reported as economic, easily accessible, flexible, and interactive.	The approach is beneficial for training a large group of students. Moreover, their study habits will be improved as a result of full-time course engagement.
**16**	Research article (Chiou, 2020) [43]	USA	North America	300	Undergraduate students	ZOOM, LabCam, and Canvas learning management system (Canvas-LMS)	PowerPoint lectures were delivered on ZOOM while the live microscope view was also shared to the students by remotely connecting the instructor’s mobile phone with the microscope using LabCam. Moreover, the sessions were also recorded and shared with the students. Tests were conducted on Canvas-LMS, and at the end, responses from students were recorded.	The results of the examinations were comparable to that of traditional learning whereas 67% of the students found the method best and interested.	The method is easy and inexpensive for microscopic learning, online lectures, and assessment.
**17**	Letters to the Editor (Cheong, Chee, Ng, & Jen, 2020) [32]	Singapore	Asia & Pacific	13	Undergraduate students	ZOOM, WebEx, and LearnHaem (https://learnhaem.com)	Microscope real-time slide images were taken and shared on online platforms during the lecture and the recorded sessions were uploaded on LearnHaem for being freely available to the students anytime from anywhere.	The evolution in learning methods was well welcomed by students.	Reduce the learning barriers during the time of the pandemic.
**18**	Letters to the Editor (Wang, Emmad, Jiayi, & Bartlett, 2021) [44]	Canada	North America	50	Postgraduate students	ZOOM, Articulate E-learning software, and IMS Cloud view	Provided daily (around 1.5 to 2 h) live lectures on ZOOM with neuroradiological case-based training using Articulate E-learning software and IMS Cloud view.	The residents observed improvement in their workflow.	Reduce the impact on radiological education and provide opportunities for medical residents to enhance their understanding and command of the course during the pandemic.
**19**	Letters to the Editor (Pacheco, Noll, & Mendonça, 2020) [51]	Brazil	South/Latin America	N/A	Undergraduate students	ZOOM, Google Meet, WhatsApp, and Athena Hub	Online sessions were conducted on ZOOM and Google Meet with weekly assessments on WhatsApp. The 3D anatomy software, Athena Hub, was also used.	Video sessions on Google Meet were more welcomed due to no time limit restriction as compared to ZOOM (40 min of time limit per session) and the use of 3D software minimized the gap to practical classes.	The innovative learning strategy is the best remote teaching alternative especially for anatomy students.
**20**	Research article (Chang, Jiang, & Xu, 2020) [35]	China	Asia & Pacific	32	Postgraduate students	CCMTV, WeChat, and ZOOM	Recorded video lectures were shared on the clinical network platform CCMTV with the students along with a time-bound assessment task. Discussion on each video was conducted on WeChat and the online ZOOM platform. Examinations were conducted on the ZOOM platform. The feedback of students regarding the quality of teaching was also recorded.	The model has been appreciated by the students and they have also found it helpful in completing their degree on time.	Although the method lacks practical learning but provides effective professional knowledge.
**21**	Research article (Hofmann, Harding, Youm, & Wiechmann, 2020) [45]	USA	North America	14	Undergraduate students	ZOOM	Virtual bedside teaching rounds were conducted on ZOOM. An iPad, connected to a computer, was used. Students connected to the videoconference observed and communicated with patients. Feedback, based on a four-point Likert scale from strongly agree to strongly disagree, was filled at the end of each round.	More than 90% of the students agreed that the adopted methodology was beneficial and improved their clinical skills. They were interested to continue the virtual rounds in the future also.	This adaptation is one of the best alternatives to traditional bedside rounds. The method allows students to remotely interact with patients.
**22**	Research letter (Nelson, Marshall, Kelly, & Vuthiganon, 2020) [46]	USA	North America	71	undergraduate students	Harbor (a learning management System of the Medical University of South Carolina) and Microsoft Teams	Evidence-based research topics were provided through Harbor along with statistical methods, literature search tools, and as well as library resources. The students were supervised by more than 30 mentors through Harbor Blackboard’s open learning management system and Microsoft Teams.	The method was found to be helpful in maintaining socially distant mentorship, and thus, the research activities have not been affected by the pandemic.	Creates virtual research opportunities through a mentorship approach to help students complete their research projects on time during the pandemic.
**23**	Research article (Naidoo, 2020) [50]	Durban	South Africa	31	Postgraduate students	Zoom, Moodle, and WhatsApp	Three interactive online mathematical workshops on academic writing, accessing, and providing feedback for algebra, and identifying misconceptions in geometry were conducted using ZOOM and Moodle, and the workshop resources, videos, and recordings were shared on an online repository. In addition, two discussion forums were accompanied at the end of workshops using WhatsApp and Moodle in which the contents of the workshop were discussed. The participants were also asked about strengths, limitations, and their experience of using the above digital platforms.	The participants reported a positive response to discussion forums on WhatsApp and encouraged the sharing of workshop resources, videos, and recordings. However, they experienced difficulties while using ZOOM and Moodle and thereby wished for proper training before using the platforms.	Use of social media platforms in digital pedagogy is worthy whereas sharing of the training resources including recorded lectures and videos can help students access the shared contents anytime and from anywhere and engage them in the course. Yet, proper training and availability of resources should be ensured for digital education.

**Table 2:** Description of the articles categorized into new applications or design of software

**S. No.**	**Article type**	**Country**	**Region**	**Number of participants**	**Type of participants**	**Online latforms**	**Description of adopted strategy**	**Outcomes**	**Strength of evidence**
**1**	Research Letter (Laurence, Fryer, Sonnier, & Taylor‐Bishop, 2020) [26]	USA	North America	20	Undergraduate students	ZOOM platform and Epidemix	Course in infection diseases was taught to the dental students in three sessions, in the form of lectures (session one and two) and through the software developed (session three) of about 1 h each to make available the infectious disease transmission visualizing and understanding to a wider audience.	More than 87% of the participants have found the course useful and improved their understanding of infectious disease modeling.	The developed software is free to all users and can be used through an easy-to-use interactive interface. Furthermore, complex courses like infectious disease modeling that require specialized mathematical training can be effectively taught with minimal training.
**2**	Research Letter (Trujillo, Tirado, Vivas, Eulufi, & Cohen, 2020) [52]	Chile	South/Latin America	190	Postgraduate students	A web-mobile based technology (LAPP)	Pre-recorded video instructions were delivered, trainees watched and uploaded exercises, and received feedback within 72 h by experts.	Participants received more than 13,000 feedback from the experts on their uploaded videos.	Convenience and easy scalability.

#### New application or software design

New application software called LAPP and Epidemix were developed to provide a user-friendly interface with minimized technology-related challenges. These applications received extremely positive user feedback [26]. In addition, the applications can be used to easily conduct complex courses.

#### Social media platform

The social media platform WhatsApp was used as a teaching tool during the pandemic in Asia and the Pacific such as Pakistan and India (see Table 3). The platform has reportedly been used effectively for both training and assessment during the pandemic [53]. However, the students identified a lack of real-world interaction and problems related to Internet connectivity and device availability as the main disadvantages of this teaching method identified in these studies.

**Table 3:** Description of the articles categorized into a social media platform

**S. No.**	**Article type**	**Country**	**Region**	**Number of participants**	**Type of participants**	**Online platforms**	**Description of adopted strategy**	**Outcomes**	**Strength of evidence**
**1**	Short Communication (Ul Bari, 2020) [33]	Pakistan	Asia & Pacific	28	Postgraduate students	WhatsApp	Students were divided into two equal groups. One group received routine physical lectures, case presentations, journal clubs, and tutorials with all PPEs and proper social distancing for two weeks. The group was self-quarantined and replaced by a second group for 2 weeks. During isolation, WhatsApp platform was used for interaction. Moreover, regular assessment was conducted in MS Word file on WhatsApp.	Feedback from the students revealed that the method was beneficial and interesting. Furthermore, 20% of the course, generally required 6 months, was learned by the students in 2 months.	The method engaged students in academics without facing any technology-related challenges. It was also proved productive and fulfilled recruitments of licensing bodies for physical training.
**2**	Letter to the Editor (Sud, Sharma, Budhwar, & Khanduja, 2020) [53]	India	Asia & Pacific	N/A	Undergraduate students	WhatsApp and Google form	PowerPoint presentations comprised of clinical cases with pictures were delivered. Additional reading materials and related clinical and surgical video procedures were shared on the WhatsApp group of the students. The online assessment on Google form was conducted for each session to engage the students and at last feedback from the students was recorded.	More than 90% of the students found online classes the best alternative to physical classes. The major benefit of the adopted strategy indicated by the students was the easy availability of the material from anywhere at any time.	Although Internet connectivity is a major barrier in online education, the method is appropriate to overcome the barrier on a temporary basis.

#### Simulation and virtual reality-based tools

Simulation and online learning based on virtual reality originated in North America such as the USA and Europe such as Hungary (see Table 4). 3D visualized technologies such as Blackboard Collaborate, Netter 3D Anatomy, 360° Virtual Operating Rooms, Manikin surgical tools, Aperia Image Scope, and Leica’s Web Viewer were used successfully to effectively deliver hands-on training remotely [29, 54, 55].

**Table 4:** Description of the articles categorized into simulation and virtual reality

**S. No.**	**Article type**	**Location**	**Region**	**Number of participants**	**Type of participants**	**Online platforms**	**Description of adopted strategy**	**Outcomes**	**Strength of evidence**
**1**	Letter to the editor (Schlégl et al., 2020) [54]	Hungary	Europe	46	Undergraduate students	Manikin surgical tools	Reproducible curriculum for distance education was established for teaching basic surgical skills (knot tying, suturing, and laparoscopic skills) to students by using tools that are easily available at home.	79% of original learning objectives were achieved completely and 15% were achieved partially by the students. Exam results were compared with the previous 2 years. Reports suggest that students were highly satisfied with the course given the circumstances.	Except for the laparoscopic suturing and special instrument handling, teaching of basic knot tying, suturing, and laparoscopic skills by using homemade tools and distance education, can be done with efficiency and a high level of student satisfaction.
**2**	Research Article (Moore, Stallard, Tittemore, & Lee, 2020) [29]	USA	North America	N/A	Undergraduate students	ZOOM, Echo360 and ExamN	Students were facilitated with recorded virtual meetings on ZOOM and Echo360. The exams were conducted using the ExamN testing solution.	More than 80% of the students were satisfied with the strategy and found it beneficial.	The use of pre-recorded content and the facility of technical support have maximized student learning during the pandemic.
**3**	Letters to the Editor (Kim, Brinster, & Meehan, 2020) [55]	USA	North America	N/A	Postgraduate students	WebEx, ZOOM, Aperio ImageScope, and Leica’s Web Viewer	The weekly sessions were conducted on ZOOM or WebEx using virtual microscopy with Aperio ImageScope or glass slide microscopy with a digital camera. The live sessions were also recorded for playback.	Ease of navigation was indicated higher on the virtual microscope than on glass slide one. Similarly, sessions conducted through virtual microscopy were found more feasible.	Aperio ImageScope platform of the virtual microscope allows changes in image magnification, rotation of the image, and annotations.

## Discussion

### Challenges

The COVID-19 pandemic has disrupted every aspect of modern society and its social fabric, especially educational institutions. It has very negative effects, especially in developing countries. As a result, it has transformed the world’s education system and forced us to redesign it. In addition, students lost internship opportunities and personal freedom, suffered personal losses, and their safety was threatened by infections and related morbidities [56].

The challenges posed by COVID-19 turned universities learning management systems and curricula upside down, particularly for various graduate programs. This has resulted in the cessation of all academic activity, reduced in-person educational opportunities, and severely restricted student access to institutions, particularly at universities where annual enrollment is mainly based on international students. The situation became increasingly unclear, with no planning and no clear end in sight, along with fears of contracting disease, a shortage of personal protective equipment (PPE) and the urgent need to maintain physical and social distancing [26, 28]. Social distancing measures forced students to be quarantined and relocated to remote settings. This also underlined the need for an alternative educational model [38, 40, 42, 49]. However, to ensure the provision of education for these students, online platforms such as video tutorials were introduced [53].

Institutions providing undergraduate and higher education had to deal with the consequences of the sudden closure of campuses, reduced hands-on attendance, diminished on-site learning environments, and the cessation of in-person classes [43, 53]. To deal with the fallout from these consequences, institutions had to develop innovative methods to ensure uninterrupted, quality education. The education system has made a rapid transition to online education, which indeed seems to be best practice under the circumstances [48]. In addition, professional graduate and postgraduate programs that offered and required internships or placements as part of their curriculum requirements had become a real problem for newcomers [57, 58].

Education before the pandemic featured a hands-on learning environment, case-based learning, and hands-on workshops. The Association of American Medical Colleges (AAMC) issued guidelines for students not to be allowed direct contact with patients who have tested positive for the coronavirus. As a result, universities prevented their students to continue hospital clerkships or other activities related to patient care [41, 45]. These strategies led to the discontinuation of various traditional learning activities due to the enforcement of physical distancing guidelines in classrooms and laboratories [51, 59]. The implementation of social distancing measures resulted in restricted access to research laboratories and restricted research on humans and animals. Retrospectives, literature reviews, and survey-based studies were the viable project options for research students. Research activities and opportunities at research institutes were also declined. The paradigm shift to e-learning was widely needed and accepted around the world. However, it could never replace the quality of learning that is mainly acquired through practical experience, such as learning instruments/equipment and troubleshooting manual skills. In addition, the transition to online education became a major challenge, with education struggling to adapt to technological innovations while COVID-19 acted as a catalyst for this slow transition [54, 60].

Most undergraduate students are computer newbies. Therefore, technology-based learning became a challenge. It was seen as a barrier to the rapid shift to online education [47]. A major challenge faced by the universities was the lack of tech-savvy human resources [29]. Online and virtual education also raised significant concerns about student assessment, as remote assessment was difficult to monitor and making remote assessment fair was therefore a difficult task [30]. In addition, poor Internet connections, lack of technical understanding, and limited resources made it difficult to provide quality education [31].

Postgraduate education was also significantly impacted by the pandemic. Theoretical learning became the only option for postgraduate medical students during the pandemic. Face-to-face classes, lectures, seminars, and conferences were reduced to a virtual environment [33]. It has become difficult for postgraduate students to continue their education within a safe environment as student health was a top priority. In addition, moving practical lectures to an online medium was a difficult task. Visiting teaching opportunities were canceled [34, 35]. Most online education platforms are expensive which puts a heavy strain on the annual budget. The surgical residency was one of the first to be affected by the pandemic and saw a sharp drop in elective surgeries with immediate effect [36]. In addition, only urgent surgeries were recommended in practice guidelines, and the cancelation of elective surgeries had a major impact on surgical resident training programs. The universities tried to meet the challenges with all available resources [61].

Social distancing strategies implemented included the use of home offices, individual reading rooms, the cancelation of conferences, and limited participation in hands-on training [37]. The University of Toronto’s Diagnostic Radiology Program, which covers eight hospitals with 50 residents, is one of the largest assistance programs in Canada and was also affected by the COVID-19 pandemic [44]. The education system was completely switched to online education, for example practical objects based on glass slide microscopy wer replaced by virtual microscopy for dermatology education [55].

On the other hand, the students were not able to understand the course of events. They did not have enough mentoring options and could not build a team relationship. These changes had a serious impact on students’ self-confidence and personality information [62, 63]. In addition, the most difficult challenge faced by teachers was to simulate the practical teaching in order to be able to demonstrate it easily. The education ministers of different countries suggested that education should not suffer, which is why educational tools based on virtual simulation were integrated into the education system [64]. Therefore, it has become necessary for institutions to adopt alternative teaching methods and explore virtual delivery of education through social media or online platforms [65].

### Solutions

Distance learning (DL) or technology-based learning (TB) is nothing new to many developing countries. The approach is used as synchronous learning based on real-time interactive lectures and asynchronous learning based on self-study and discussion in various forums such as emails [66, 67]. Video conferencing has been used in education since the 1960s [68]. Flexibility, accessibility, reduced costs, portable learning materials, self-based learning, time efficiency, and reduced risk are some of the stated benefits of online learning. Even before the current pandemic, online platforms are being used by various institutions around the world, especially in developed countries [69, 70], showing that online education as an academic norm is not only associated with the COVID-19 pandemic and several online education-related video-conferencing software have been used, including Zoom, Skype, and Cisco Webex [71, 72].

Zoom turned out to be the most widely used video-conferencing platform for synchronous education, which offers useful features for smooth communication, such as these include a chat board, a hand-raising system that increases student attention and engagement, screen sharing that allows presenters to view slides, and the recording of lectures for later viewing [34, 73]. It could be observed that not only the students, as a generation with an affinity for social media, accepted this form of learning, but also that the feedback from the faculty members in a study showed that, despite the use of new teaching methods, there was a significant degree of satisfaction with online teaching ruled [74].

Distance learning provided an opportunity for educators to develop a close connection and dialogue with students, particularly those with intellectual disabilities and learning difficulties [51]. Student reviews were mixed, with some advocating a blended strategy for the post-pandemic era, others expressing a negative opinion of online learning, calling it an unsuitable mode, particularly for medical education, and still others expressing the need for one comprehensive training to adopt the online mode learning. Studies have shown that most students favor hybrid learning because it overcomes some traditional teaching barriers but requires appropriate teacher training and institutional support [74]. The flipped classroom is a common pre-pandemic approach and can therefore be used for asynchronous and synchronous lessons [75]. Virtual meetings were found to be more engaging for students as they have the feel of a live presentation, and online video lectures and webinars are expected to continue post-pandemic due to greater international exposure and lower costs [61, 76].

It is believed that while providing valuable content to students, most online education platforms lack the ability to provide economically personalized feedback. For this reason, a web-based mobile platform called LAPP was set up. This application connects students with tutors remotely via their phones. Students can submit their exercises and receive feedback via video, drawing, audio, or text content. Almost 3700 replies were reported as successfully submitted. Another application, Epidemix, was developed to provide security and convenience for teachers and students. The applications can be used on a user-friendly interactive interface or on a mobile phone and are free for all users [26].

Social media is a tool that allows information to be shared in various formats, including videos [77]. There has been an increasing trend to use social media for teaching and learning purposes, and a number of social media platforms such as Facebook, WhatsApp, and Instagram have been used for collaborative learning and better communication [33]. A preference for “YouTube” for technical lectures can already be seen in the literature, as videos are a useful, expressive, and easily accessible information tool [77].

YouTube is the most popular source of tutorial videos because of its free content, ease of use, and familiarity among web users [77]. However, an evaluation of YouTube videos revealed that more than 95% of the videos watched by students for learning purposes were of poor educational quality. Therefore, it has been suggested that academics must conduct a critical evaluation of YouTube videos before proposing them to students [33, 53, 78]. Likewise, the Telegram application is considered a valuable mobile application with numerous utilities and many helpful features, including easy access to educational videos, no file size or file format limitation, unlimited member capacity, good connectivity and security, and subscriptions to journals and e-books without the need a browser or website support. Some limitations such as limited group member capacity and file-sharing ability have been found to be associated with applications such as WhatsApp and Facebook [79]. Although these two applications are considered to be widely used platforms worldwide that offer free services. The applications provide opportunities for knowledge sharing, assessment tools, and cognitive enhancement, even when resources are scarce [33]. WhatsApp is reported to have been used effectively for both training and assessments during the pandemic. However, students cited a lack of real-world interaction and problems related to Internet connectivity and device availability as the main disadvantages of this teaching method [53].

The main advantages of using social media platforms for teaching include avoiding wasted time, easy curriculum coverage, standardized assessments, preparing and conducting exams with the active participation of students, economic feasibility, familiarity with technology, and formative assessments continue academic education in an effective manner [33, 53]. However, the few inherent disadvantages include the lack of hands-on training and real-time skills-based learning, the risk of cheating during an online assessment, and limited experience with a variety of real-world scenarios [33].

Simulation-based training has been suggested as the main solution for training practical skills [52]. Various simulation techniques have been developed or used. These techniques are often based on virtual reality such as the LapSim simulator, are based on 3D models such as silicon 3D simulation kits, or are based on real-time hands-on experiences such as head-mounted camera devices and whole slide imaging [62, 80–83]. Although many of the simulation-based platforms are considered quite expensive and require a fast Internet connection, they allow learners to see and interact with the lecturer and offer the best alternative for technical students during the period of social distancing [62, 80].

### Strengths and limitations

The strength of the present study is in providing an in-depth overview of the strategies implemented by different universities in response to the educational challenges during the pandemic. It also summarizes the innovative solutions adopted in the selected studies to reduce the educational gap at the time of COVID-19. The research articles generated from the databases were included, such as editorials, letters, and letters to the editors. However, there may be some limitations, for example, the data obtained from selected databases did not contain information on educational approaches for other programs, such as graphic design, engineering, and visual studies in art, but mainly related to medical education. The selected studies are unstructured and show large design variations. The selection criteria were limited to undergraduate and postgraduate education. The main source of the scientific database used was PubMed, EMBASE, and supplemental searches were provided by Google Scholar.

### Remarks

The present study offers practical and experienced solutions adopted by several universities from both developed and underdeveloped countries for the implementation of online education. Although online learning cannot replace hands-on learning and also has many limitations, it can be used effectively for study continuation in an uncertain period. Our goal was to explore the best alternative method available for the continuation of the education system, especially for college or university students, and to bridge the gap between students and education during the time of the pandemic. There has been much criticism of online education and related technologies, but the availability of these technological advances represents the only alternative solution to combating the education crisis caused by a pandemic.

Means of communication such as interactive video tools, digitized content, visual media, and other web-based platforms have proven to be efficient training and learning tools in the learning process, regardless of time and place [84]. There are various social media tools like WhatsApp, Telegram, YouTube, Facebook, Skype, Twitter, Snapchat, and Instagram and other platforms like ZOOM, Google Classroom, Google Meet, Microsoft Team, GoToMeeting, WebEx, and Adobe Connect, which have proven valuable in delivering distance learning [56, 77, 79, 85, 86]. But certain challenges have been observed, in particular the need for digital devices such as smartphones, laptops, or desktops and the requirement for an uninterrupted Internet connection [87]. Affordability of such electronic or digital devices for people from countries with low-income economies and a per capita income of less than $1050, where basic needs such as food, clothing, and shelter are already the greatest problems, such as in Somalia, Sudan, Afghanistan, Yemen, Tajikistan, and Guinea, is another challenge that needs to be addressed [88].

In addition, the lack of electricity, which is a prerequisite for functioning electronic devices, poses an additional challenge in the least developed countries. The lack of literacy skills and the availability, accessibility, or affordability of the Internet have also been considered insignificant in these regions as classified [89]. Affordability of digital devices should be ensured and Internet connectivity should be restructured by governments.

People in countries with middle economies like India, Pakistan, China, and Brazil have access to electronic devices to some extent, but uneconomical online platforms, lack of digital literacy, and lack of strong Internet infrastructure have been reported as the main barriers to online education [33, 53, 89, 90]. Social media platforms such as WhatsApp, Facebook Live, YouTube, Twitter, Snapchat, and Instagram as well as free or low-cost video conferencing tools such as Microsoft Team and ZOOM have been widely used as distance learning platforms in these regions [20, 32, 33, 44, 56, 58, 85]. As problems with Internet availability and connectivity were recognized as major obstacles in these countries, recorded video lectures were more welcomed. Although online education continued in these countries, the lack of trained staff and limited Internet services in remote areas meant that students could not properly access the live or recorded lectures [47]. However, it has been found that students in these regions favor a hybrid or blended education system [33, 53, 89, 90].

The countries with high-income economies like the USA, Australia, Singapore, Japan, Korea, the UK, Ireland, Italy, Hungary, Canada, Chile, and Mexico have succeeded in developing effective online virtual simulation education based on 3D devices such as Echo360, LabCam, Netter 3D Anatomy, Canvas-LMS [80, 81]. The platforms, based on a 360-degree virtual operating room, are essential for an education system where learning is almost based on practice rather than theoretical knowledge [20, 55]. These digital devices and the new technologies that accompany them require practical skills and training to be able to use them. According to the UNESCO report, nearly 750 million adults still lack basic literacy skills. Therefore, digital skills and literacy pose a challenge not only for least developed countries but also for developed countries [15]. In addition, although these countries have advanced digital infrastructure, inequalities in access to technology access are reported, such as in the USA, where access to technology is divided by race, income, and geography [88]. Video conferencing tools with interactive online lectures and training courses on new technologies are needed. Although hands-on experience is irreplaceable, adapting to technological changes can help overcome some problems of online education [77].

This pandemic also teaches us the lesson that international organizations like the WHO should emphasize building the online education infrastructure to prevent future education crises. The role of students, teachers, and parents must be clearly defined in distance learning to foster future outcomes. Online tools and courses should be evaluated regularly, while program learning outcomes should be assessed based on attitudes, skills, and knowledge. Easy access to technology and the Internet is seen as a key solution to overcoming the main weaknesses of online education [91]. In doing so, excess funds for governments and institutions must be vided by international fundraising organizations, especially for low- and middle-income countries, poor students, and digitally divided areas of high-income countries, to ensure their easy access. Teachers should be properly trained and an appropriate suitable assessment system for student achievement should be developed to improve the quality of education and reduce difficulties in assessing student learning outcomes. A global organization for cooperation and harmonization between institutions should be developed to provide quality and impartial education to students around the world.

## Conclusion

The online education system is a practical and voluntary choice of many education systems but has now become mandatory for everyone, especially in the higher education system. The review summarizes common teaching issues and possible solutions faced by institutes/universities in countries classified into different income economy groups during the pandemic. Poor Internet infrastructure, power outages, and limited resources were the main challenges for the countries with the lowest- and middle-income economies, while a lack of technical knowledge of novel virtual tools and simulation techniques or devices was the most common problem for the countries in the high-income group. Recorded video lectures and heterogeneous systems of online and physical education classes are considered effective methods to reduce technology-based challenges. However, online platforms have proven to be a powerful tool for educators, allowing them to continue teaching in times of social isolation and ensure social connectivity while maintaining physical distance. Among these, social media platforms proved to be the best tools for distance learning. ZOOM has been one of the most widely used online platforms in both developed and underdeveloped countries. Although the world is digitizing day by day and innovative technologies are being developed rapidly. It is concluded that basic knowledge is also essential for using these new technologies. Therefore, countries need to invest more in online education training and ensure adequateInternet accessibility and availability, especially in remote areas. Although these available platforms have been successfully adopted as an alternative learning method and the recorded video lectures have received more positive feedback from students, appropriate training facilities, and adequate learning opportunities exist in all regions of the world, especially in remote areas, with availability and affordability of resources should be ensured.

### Supplementary Information

**Additional file 1:** **Table S1.** PRISMA-ScR checklist.

## Data Availability

The datasets used and/or analyzed in the present study are available from the corresponding author on reasonable request.

## Abbreviations

COVID-19: Coronavirus disease 2019
WHO: World Health Organization
UNICEF: United Nations International Children’s Emergency Fund
UNESCO: United Nations Educational, Scientific and Cultural Organization
GDP: Gross domestic product
PRISMA-ScR: Preferred Reporting Items for Systematic Review and Meta-Analyses extension for Scoping Reviews
SARS-CoV-2: Severe acute respiratory syndrome coronavirus 2
AAMC: Association of American Medical Colleges
DL: Distance learning
TB: Technology bases
